# Evaluation of oral cavity squamous cell carcinoma undergoing selective neck dissection with positive nodes among Indian patients

**DOI:** 10.6026/9732063002001800

**Published:** 2024-12-31

**Authors:** Salini Kumari Dash, Asutosh Pradhan, Madhurima Rudra, Ravi Kumar, Sushil Kumar Sahoo, Manisha Mohanty

**Affiliations:** 1 Department of Oral and Maxillofacial Surgery, Hi-Tech Dental College and Hospital, Bhubaneswar, Odisha, India; 2 Department of Allied Health science, Sister Nivedita University, Kolkata, West Bengal, India; 3 Department of Dentistry, Heritage Institute of Medical Sciences, Varanasi, Uttar Pradesh-221005, India; 4 Private Consultant, Bhubaneswar, Odisha, India

**Keywords:** Oral cavity squamous cell carcinoma, selective neck dissection, node-positive, disease-free survival, overall survival, regional recurrence

## Abstract

Selective neck dissection (SND) is commonly performed in patients with node-positive oral cavity squamous cell carcinoma (OCSCC) to manage regional metastasis while minimizing morbidity. Therefore, it is of interest to evaluate the effectiveness of SND in patients with node-positive OCSCC. We conducted a retrospective cohort study including 150 patients diagnosed with node-positive OCSCC who underwent selective neck dissection. Patient demographics, tumor characteristics, surgical outcomes and adjuvant therapies were recorded. The primary endpoints were disease-free survival (DFS) and overall survival (OS). Secondary endpoints included regional recurrence and postoperative complications. Selective neck dissection is a viable surgical option for patients with node-positive OCSCC, providing acceptable regional control and survival outcomes. Extra capsular spread and positive surgical margins are significant prognostic factors for recurrence. Careful patient selection and meticulous surgical technique are essential for optimizing outcomes.

## Background:

Oral cavity squamous cell carcinoma (OCSCC) is one of the most common head and neck cancers, accounting for approximately 90% of all malignancies in this region [1]. The presence of lymph node metastasis is a well-known adverse prognostic factor, significantly reducing both overall survival (OS) and disease-free survival (DFS) in affected patients [2, 3]. Management of the neck in patients with node-positive OCSCC is critical, as regional metastasis is often the first site of recurrence and a major cause of treatment failure [4]. Selective neck dissection (SND) has emerged as a popular surgical approach for treating patients with clinically or pathologically positive lymph nodes. Unlike comprehensive neck dissection, SND aims to remove only the lymphatic levels most at risk for metastasis, thereby reducing postoperative morbidity while maintaining oncologic efficacy [5]. Previous studies have demonstrated that SND can provide acceptable oncological control in patients with early-stage and selected node-positive head and neck cancers [6, 7]. However, the long-term outcomes of SND in node-positive OCSCC remain a topic of debate, particularly with respect to regional control, recurrence rates and survival outcomes. Therefore, it's if of interest to evaluate the efficacy of selective neck dissection in patients with node-positive OCSCC, focusing on survival outcomes and factors associated with regional recurrence.

## Materials and Methods:

This retrospective cohort study was conducted at a tertiary care center, reviewing the medical records of patients diagnosed with node-positive oral cavity squamous cell carcinoma (OCSCC) who underwent selective neck dissection (SND). The study was approved by the Institutional Review Board and the requirement for informed consent was waived due to the retrospective nature of the study.

## Patient selection:

Patients were included if they met the following criteria:

[1] Histologically confirmed OCSCC.

[2] Clinically or pathologically node-positive (N1-N3) disease.

[3] Underwent selective neck dissection as part of their primary surgical treatment.

[4] No prior treatment for head and neck cancer.

[5] Adequate follow-up data for at least 12 months or until death.

Patients with distant metastasis at presentation, synchronous primary tumors, or those who received non-surgical treatment as their primary modality were excluded from the study.

## Data collection:

Data collected included patient demographics (age, sex), tumor characteristics (tumor size, location, histological grade and T stage), nodal status (number of nodes involved, presence of extra-capsular spread), surgical details (extent of neck dissection, margin status), adjuvant therapy (radiotherapy, chemotherapy) and follow-up information (recurrence, survival outcomes and complications).

## Surgical procedure:

Selective neck dissection was performed according to the levels of lymph node involvement. Levels I-III was typically dissected for oral cavity tumors, with levels IV and V included if clinically indicated. The decision regarding the extent of neck dissection was based on the preoperative imaging and intraoperative findings.

## Outcome measures:

The primary outcomes were disease-free survival (DFS) and overall survival (OS). DFS was defined as the time from surgery to the first occurrence of local, regional, or distant recurrence or death from any cause. OS was defined as the time from surgery to death from any cause. Secondary outcomes included regional recurrence rate and postoperative complications, such as wound infection, seroma and shoulder dysfunction.

## Statistical analysis:

Statistical analyses were performed using SPSS software (version 23). Survival curves were estimated using the Kaplan-Meier method and compared using the log-rank test. Cox proportional hazards regression was used to identify factors associated with DFS and OS. A p-value of less than 0.05 was considered statistically significant.

## Follow-up:

Patients were followed up at regular intervals postoperatively, with clinical examinations and imaging studies performed as per institutional protocol. The median follow-up period was 36 months, ranging from 12 to 120 months. Recurrences were confirmed by biopsy and/or imaging studies.

## Results:

A total of 150 patients with node-positive oral cavity squamous cell carcinoma (OCSCC) who underwent selective neck dissection (SND) were included in the study. The median age at diagnosis was 62 years (range: 35-85 years), with a male-to-female ratio of 2:1. The majority of patients had tumors located in the tongue (40%) and floor of the mouth (30%) (Table 1).

## Survival outcomes:

The median follow-up period was 36 months (range: 12-120 months). The 3-year disease-free survival (DFS) rate was 68% and the overall survival (OS) rate was 75%. Regional recurrence occurred in 30 patients (20%), with a median time to recurrence of 18 months (range: 6-48 months) (Table 2).

## Postoperative complications:

Postoperative complications were recorded in 35 patients (23%). The most common complications included wound infection (8%), seroma (5%) and shoulder dysfunction (10%) (Table 3).The findings suggest that selective neck dissection provides acceptable regional control in node-positive OCSCC with a manageable rate of postoperative complications.

## Discussion:

Selective neck dissection (SND) has become an increasingly preferred surgical approach for managing node-positive oral cavity squamous cell carcinoma (OCSCC) due to its potential to reduce morbidity while maintaining effective regional control [1, 2]. Our study demonstrated a 3-year disease-free survival (DFS) rate of 68% and an overall survival (OS) rate of 75%, which are comparable to the outcomes reported in previous studies investigating the efficacy of SND in node-positive head and neck cancers [3, 4]. The regional recurrence rate observed in our cohort was 20%, with the majority of recurrences occurring within the first two years postoperatively. This finding aligns with the literature, which suggests that the highest risk of recurrence in OCSCC patients is within the initial two years following treatment [5, 6]. Factors such as extra capsular spread (ECS) and positive surgical margins were identified as significant predictors of poor DFS in our multivariate analysis, consistent with previous research indicating that ECS is a crucial prognostic factor for regional recurrence and overall survival in patients with head and neck squamous cell carcinoma [7, 8]. Extra-capsular spread (ECS) is known to significantly impact prognosis, with several studies demonstrating its association with increased rates of regional recurrence and decreased survival [9, 10]. The presence of ECS indicates a more aggressive disease course and its identification often necessitates the use of adjuvant therapy, such as chemo radiotherapy, to improve loco regional control [11, 12]. In our study, ECS was present in 35% of patients and it was a significant predictor of poor DFS, with a hazard ratio of 2.5.

Positive surgical margins have also been well-documented as an adverse prognostic factor, increasing the likelihood of local recurrence and reducing overall survival [13, 14]. Achieving clear margins is crucial in the surgical management of OCSCC, as positive margins often necessitate additional adjuvant treatment and are associated with worse outcomes [15, 16]. Our study found a 10% rate of positive margins, which was significantly associated with decreased DFS. The overall complication rate in our study was 23%, with wound infection and shoulder dysfunction being the most common. This is in line with previous reports indicating that while SND has a lower morbidity profile compared to more extensive neck dissections; it still carries a risk of complications, particularly when performed in conjunction with adjuvant therapies [17, 18]. Shoulder dysfunction is a known complication of neck dissection, often resulting from injury to the spinal accessory nerve, which highlights the importance of careful surgical technique and postoperative rehabilitation [19, 20]. Despite the promising outcomes associated with SND, careful patient selection remains essential. The decision to perform SND should consider tumour stage, nodal burden and other patient-specific factors to optimize outcomes and minimize the risk of recurrence [21, 22]. Additionally, the use of adjuvant therapy, particularly in cases with high-risk features such as ECS or positive margins, plays a crucial role in improving loco regional control [23, 24]. Our study adds to the growing body of evidence supporting the role of SND in the management of node-positive OCSCC. However, it is important to acknowledge the limitations of this study, including its retrospective nature and the potential for selection bias. Further prospective studies are warranted to validate these findings and to establish standardized guidelines for the use of SND in this patient population [25].

## Conclusion:

Selective neck dissection (SND) appears to be an effective surgical approach for managing node-positive oral cavity squamous cell carcinoma (OCSCC), offering satisfactory disease-free survival and overall survival rates. This data shows a 3-year DFS rate of 68% and an overall survival rate of 75%, which are in line with outcomes from existing literature.

## Figures and Tables

**Table 1:** Summarizes the baseline characteristics of the study population.

**Characteristic**	**Value**
Median Age (years)	62 (range: 35-85)
Gender (Male: Female)	2:01
Primary Tumor Site	Tongue (40%), Floor of Mouth (30%)
Tumor Stage (T1/T2/T3/T4)	20% / 45% / 25% / 10%
Nodal Stage (N1/N2/N3)	50% / 40% / 10%
Histological Grade	Well-differentiated (30%), Moderately differentiated (50%), Poorly differentiated (20%)
Extra-capsular Spread	Present (35%), Absent (65%)
Positive Surgical Margins	10%
Adjuvant Therapy	Radiotherapy (70%), Chemoradiotherapy (20%)

**Table 2:** Survival Outcomes

**Outcome**	**Value**
Median Follow-Up (months)	36 (range: 12-120)
3-Year DFS Rate	68%
3-Year OS Rate	75%
Regional Recurrence Rate	20%
Median Time to Recurrence (months)	18 (range: 6-48)

**Table 3:** Postoperative Complications

**Complication**	**Incidence (%)**
Wound Infection	8%
Seroma	5%
Shoulder Dysfunction	10%
Hematoma	2%
Chyle Leak	1%
